# Crohn’s Disease in a Patient With Bardet-Biedl Syndrome: Random Anomaly or Rare Phenotypic Trait?

**DOI:** 10.1097/PG9.0000000000000333

**Published:** 2023-06-26

**Authors:** Margot L. Zuidhof, Tim G.J. de Meij, Sietse Q. Nagelkerke, Anne M. Smets, Ilan J.N. Koppen

**Affiliations:** From the * Department of Pediatric Gastroenterology and Nutrition, Emma Children’s Hospital, Amsterdam UMC, University of Amsterdam & VU University, Amsterdam, the Netherlands; † Department of Pediatric Immunology, Rheumatology and Infectious Disease, Emma Children’s Hospital, Amsterdam UMC, University of Amsterdam, Amsterdam, the Netherlands; ‡ Department of Radiology and Nuclear Medicine, Amsterdam UMC, University of Amsterdam, Amsterdam, the Netherlands.

**Keywords:** Bardet-Biedl syndrome, BBS, BBS7, Crohn disease, IBD, inflammatory bowel disease, abdominal pain, gastroenterology

## Abstract

Bardet-Biedl syndrome (BBS) is an autosomal recessive multisystem nonmotile ciliopathy. There are anecdotal reports of the co-occurrence of BBS and autoimmune diseases, including inflammatory bowel disease (IBD). We present the first case report of a child with *BBS7* who developed Crohn disease, adding to the evidence on the association between BBS and IBD. A 13-year-old girl with *BBS7* presented with abdominal pain and significant weight loss (−13%), but without other classical symptoms of IBD, such as diarrhea and blood loss. Fecal calprotectin was elevated, but on gastroscopy and colonoscopy, no macroscopic abnormalities were found. Ultrasound and MRI revealed an intestinal stenosis which was treated surgically. Histopathological examination confirmed the diagnosis Crohn disease. In conclusion, the reported co-occurrence of BSS and autoimmune diseases and the atypical presentation of IBD in this patient warrant a low threshold to perform diagnostic tests for IBD in patients with BBS and gastrointestinal symptoms.

## INTRODUCTION

Bardet-Biedl syndrome (BBS) is a rare autosomal recessive multisystem nonmotile ciliopathy, with an estimated prevalence of 0.7/100 000 people in Europe (1). BBS is characterized by a high genetic heterogeneity (2). Twenty-one different mutations have been described in BBS genes so far, with *BBS1* and *BBS10* being the most common (2). Due to heterogeneity and pleiotropy, phenotypic traits are highly variable. BBS is characterized by intellectual disability, retinitis pigmentosa, postaxial polydactyly, kidney disease (structural anomalies, hydronephrosis), and an enhanced susceptibility of developing obesity (3). Other possible features include neurodevelopment delay, ataxia, poor coordination, epilepsy, anosmia, and gastrointestinal abnormalities (including Hirschsprung and celiac disease) (3). Moreover, there are anecdotal reports of the co-occurrence of BBS and autoimmune diseases, such as autoimmune thyroiditis and inflammatory bowel disease (IBD) (4,5). In the Clinical Registry Investigating Bardet-Biedl Syndrome (CRIBBS), which consists of 743 patients with BBS (including various BBS genes), IBD was described in 9 cases (1.2%), this involved ulcerative colitis in 7 of 9 patients (6). To date, only 1 pediatric case with co-occurrence of BBS and Crohn disease has been described in the literature, this was a child with *BBS10* (4). Here, we present a second case of BBS and Crohn disease, in a 13-year-old girl with *BBS7*.

## CASE PRESENTATION

A 13-year-old girl with BBS type 7 was referred to the pediatric gastroenterologist for chronic recurrent abdominal pain. She was born at the gestational age of 34 weeks, with hexadactyly of fingers and toes and a sinus urogenitalis. At the age of 6 months, mild asymmetric kidney function distribution was established on a renogram. During the annual follow-up, her kidney function remained stable. At the age of 10 months, she started to gain weight and deviated from her growth curve. Because of these multiple problems, genetic testing was performed at 4 years of age, revealing a *BBS7* mutation, compatible with BBS.

At 13 years of age, she presented with weekly periumbilical abdominal pain during the preceding 4 months, lasting approximately 1 hour. She did not use any medication at the moment of presentation. No relation with food or defecation was noticed and no episodes of fever were reported. She defecated once a day and had yellow-colored loose stools (Bristol Stool Chart type six), without blood. Within 3 months, she had suffered significant unintentional weight loss of 5.5 kg (body weight: 43.5 kg [+1.8 SD] to 38 kg [+0.7 SD], −13%). Until the age of 12 years, her height had steadily developed between −1 and −2 SD, but at 13 years of age, she presented with stunted growth (height: 145 cm [−2.6 SD]). Laboratory testing showed an elevated C-reactive protein level (95 mg/L), low serum albumin (33 g/L), and borderline anemia (hemoglobin 6.3 mmol/L, Mean Corpuscular Volume 64 fL) without signs of iron depletion (ferritin 4.9 µg/L, Fe 4.1 mmol/L). Transaminases, bilirubin levels, kidney function, and electrolytes were all normal and a urinary tract infection was ruled out. Abdominal ultrasonography showed thickening of the terminal ileum wall and signs of stenosis with prestenotic bowel dilatation. Additionally, the intrahepatic common bile duct was dilated (9 mm) without the presence of obstructing bile stones. The patient was admitted and antibiotics were started and stopped after 3 days when results of fecal bacterial cultures returned negative. Because the fecal calprotectin level was elevated (>6000 µg/g), a gastroscopy and colonoscopy were performed to exclude Crohn disease. No macroscopic abnormalities were found. Histology of the biopsies of the ileum showed mild inflammation with the presence of microgranulomas, the colonic biopsies revealed mild, chronic inflammation. Abdominal MRI confirmed 2 stenotic segments of the terminal ileum (See Supplemental Digital Content 1 Figure, http://links.lww.com/PG9/A119) and dilatation of the intrahepatic bile ducts (See Supplemental Digital Content 2 Figure, http://links.lww.com/PG9/A120 and Supplemental Digital Content 3 Figure, http://links.lww.com/PG9/A121), without signs of primary sclerosing cholangitis. Based on the combination of clinical, biochemical, histological, and imaging findings, the diagnosis of Crohn disease was established. Initially, treatment with infliximab was proposed to reduce the inflammation contributing to the stenosis. According to protocol, the patient was screened for latent tuberculosis before initiating infliximab treatment and the Interferon Gamma Release Assay and tuberculin skin test turned out to be positive. Therefore, treatment with infliximab could not be started. Due to the severity of the symptoms and the structuring disease of the terminal ileum, treatment could not be postponed and a surgical resection of the stenotic ileal segments was performed. Abdominal tuberculosis was excluded by negative tuberculosis tests (Auramine staining, polymerase chain reaction, and culture of resected ileal tissue were negative). Treatment for latent tuberculosis was initiated with rifampicin and isoniazid for a duration of four months in total. After surgery, the patient was put on a Crohn disease exclusion diet to prevent relapse, with good effect. The abdominal pain resolved and her weight gradually returned to normal (+1.2 SD). To prevent recurrence of strictures, aside from continuing Crohn disease exclusion diet, maintenance treatment with infliximab (in combination with methotrexate) was initiated when the treatment for latent tuberculosis was completed, 6 months after surgery.

## DISCUSSION

This is the first published report of a child with *BBS7* and a concomitant diagnosis of Crohn disease. There were no macroscopic abnormalities on both gastroscopy and colonoscopy and no classical symptoms of IBD, such as diarrhea and blood loss. Eventually, ultrasound and MRI revealed an intestinal stenosis, which was treated surgically. Histopathological examination of the resection specimen eventually confirmed the diagnosis of Crohn disease and targeted therapy was initiated.

Previous case reports have described the co-occurrence of BBS and autoimmune diseases, IBD specifically, mostly in adult *BBS1* and *BBS10* patients (4,5). In line with these findings, a single case report of Crohn disease in a child with *BBS10* has been published before. In that case, unlike our case, deep ulcerations were shown on both gastroscopy and colonoscopy (4). A satisfactory explanation for the dilated bile duct was not found in our case; liver enzymes and liver function were normal. Follow-up will focus on possible development of primary sclerosing cholangitis.

The data from the CRIBBS database provided some additional information about BBS patients with IBD. Of the 7 BBS patients who were diagnosed with ulcerative colitis, 3 were male and 1 was diagnosed at the age of 3 years (sex unknown). Two BBS patients developed Crohn disease, they were both male and one was diagnosed at the age of 15 years. The other patient was 20 years old. Unfortunately, other characteristics such as symptoms at diagnose and disease characteristics were not available from the CRIBBS database. In the future, it would be valuable to strive to provide such an overview, to gain more insights into the underlying pathophysiology.

The prevalence of IBD has been shown to be significantly higher in patients with BBS (1.51%) compared with the general population (0.40%), with an odds ratio of 3.83 (5). This increased prevalence is not fully understood, it may be secondary to the inflammatory state that is seen in obesity, which is common in BBS or it may be directly related to the impaired function of the BBsome transport complex (5). The BBsome consists of 8 BBS proteins, which form a transport complex (5). The BBsome has been suggested to play a role in regulating T-cell signaling and is required for Sonic Hedgehog signaling, which regulates, among other processes, T-cell development and differentiation (5). Furthermore, the BBSome regulates trafficking of the leptin receptor, and leptin is known to act as a pro-inflammatory cytokine (5).

In conclusion, there is a potential association between IBD and BBS, which should be taken into account when evaluating patients with BBS and abdominal pain, particularly when other symptoms compatible with IBD, such as weight loss and diarrhea, are present. The atypical presentation in our patient, without macroscopic abnormalities on endoscopy, warrants a low threshold to perform diagnostic tests for IBD, including fecal calprotectin and abdominal ultrasound, in patients with BBS with gastrointestinal symptoms.

## ACKNOWLEDGMENTS

Informed consent for the publication of this case report was obtained from the legal guardians. We thank the parents for providing consent for the publication of this case report. We also thank the Clinical Registry Investigation Bardet Biedl Syndrome, Marshfield Clinic Research Institute for their cooperation and for sharing anonymized data.

## Supplementary Material
