# Supplementary figures and images for: Crohn’s Disease in a Patient With Bardet-Biedl Syndrome: Random Anomaly or Rare Phenotypic Trait?
Source: JPGN Rep. 2023 Jun 26;4(3):e333. doi: 10.1097/PG9.0000000000000333 (PMC10435024; doi:10.1097/PG9.0000000000000333)

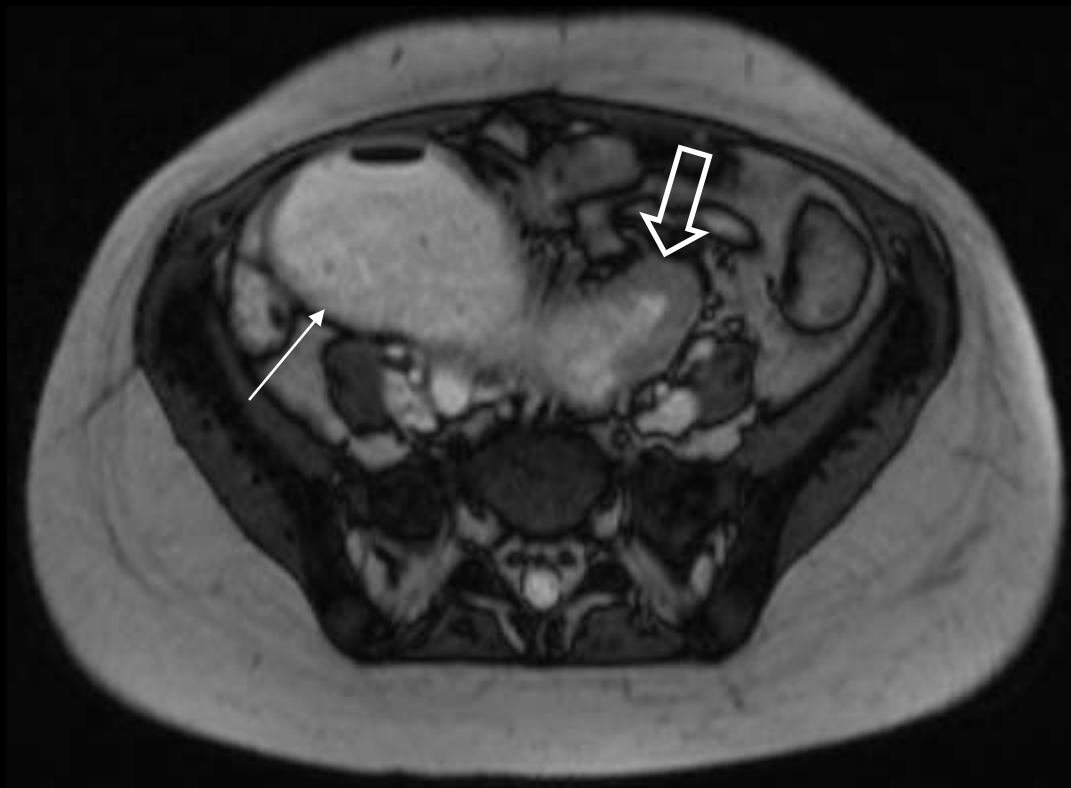

Supplement: Supplementary file 1 [file pg9-4-e333-s001.pdf]

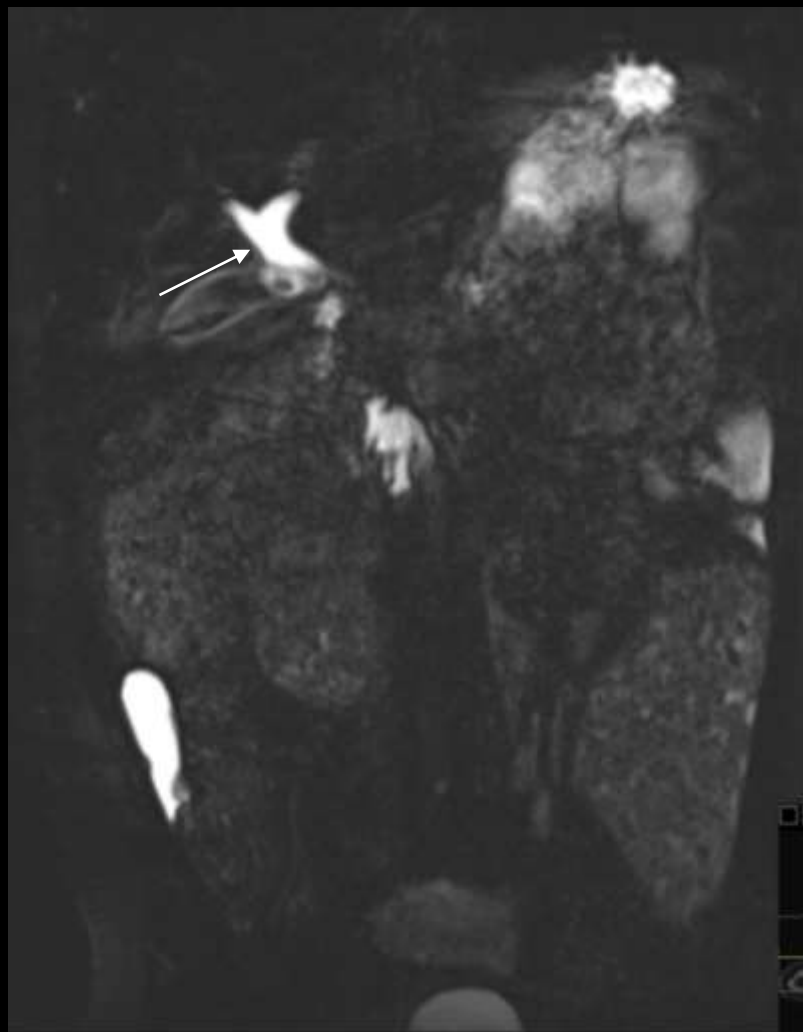

Supplement: Supplementary file 2 [file pg9-4-e333-s002.pdf]

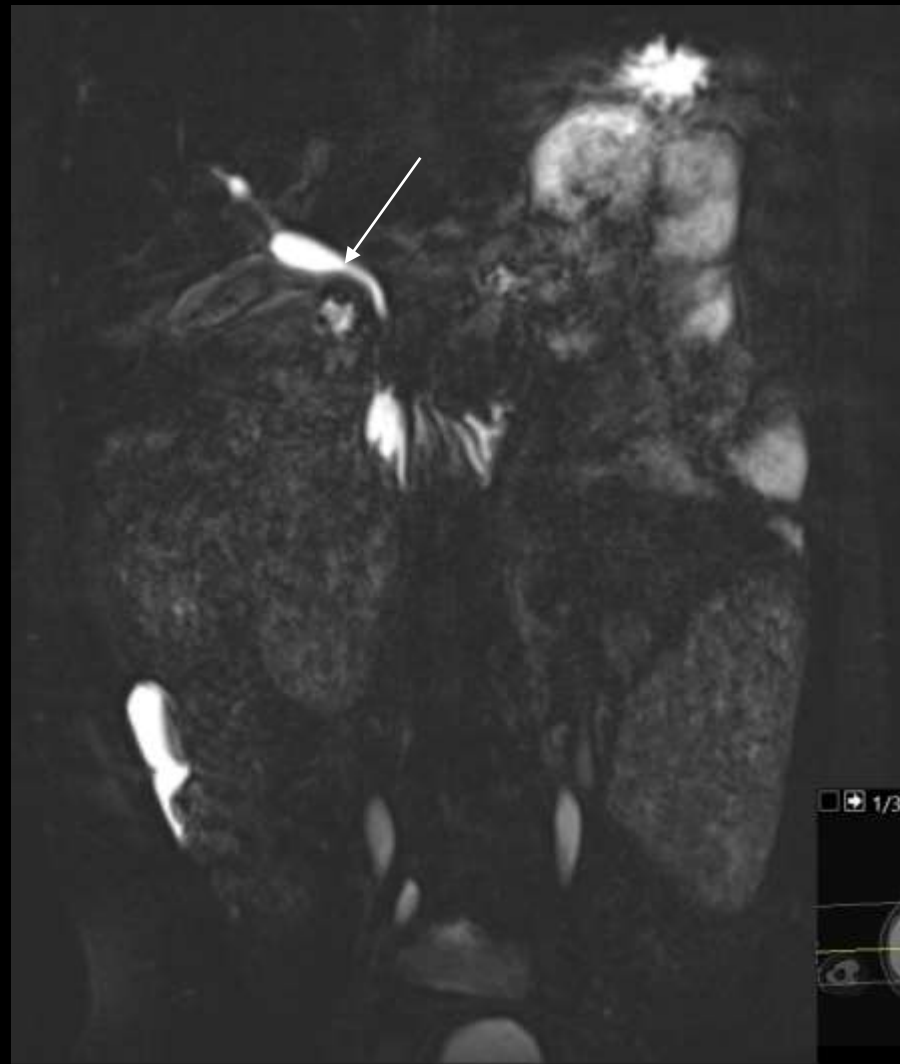

Supplement: Supplementary file 3 [file pg9-4-e333-s003.pdf]
